# The Early Diagnosis of Pulmonary Tuberculosis1Read at the meeting of the North Carolina State Medical Society at Wilmington, June 10-14, 1902.

**Published:** 1902-09

**Authors:** Silvio Von Ruck

**Affiliations:** Asheville, N. C., Laryngologist and Associate Medical Director of the Winyah Sanatarium


### BUFFALO MEDICAL JOURNAL.

VOL. XLII.—LVIII. SEPTEMBER, 1902. NO. 2.

### ORIGINAL COMMUNICATIONS.

### The Early Diagnosis of Pulmonary Tuberculosis.[1]

By SILVIO von RUCK, M. D., Asheville, N. C.,

Laryngologist and Associate Medical Director of the Winyah Sanatarium.

EVERY intelligent physician seeks primarily to determine the nature of the affection with which he has to deal in a given case before deciding upon this, that, or the other therapeutic means for its amelioration or cure. He realises also, that in many instances, the earlier the diagnosis is made the greater are the prospects of obtaining favorable results from treatment, and he further knows that sometimes, if prompt measures are not resorted to, the patient's chances for recovery may be forfeited. We are all quick to acknowledge the force of these truths in acute infectious diseases like diphtheria, and tetanus, for example, as well as in various other affections but unfortunately it must be admitted that not every practitioner so thoroughly appreciates the vital importance of an early diagnosis in pulmonary tuberculosis.

It is a well-established fact that pulmonary tuberculosis, in its early stages, is a very curable affection, but there does not as yet exist a conformity of opinion as to just what constitutes the truly early stages.

It is the purpose of this paper to consider the more incipient changes which occur in the lungs in relation to the signs and symptoms to which they give rise, and to distinguish this period of the disease from later ones in which the diagnosis is readily made, in which destructive alterations are in process or have already taken place, and in which the affection is more properly termed phthisis or consumption.

---

1. Read at the meeting of the North Carolina State Medical Society at Wilmington, June 10-14, 1902.

Various writers describe a "pretuberculous stage"[1] a prodromal period, so to speak, during which the prospective subject of pulmonary tuberculosis presents slight symptoms which often seem so inconsequent as to pass unnoticed or to be ignored. This term "pretuberculous" is both a misleading and a most unfortunate one, and the sooner it is dropped the better, for not only is there in reality no such thing as a "pretuberculous stage," inasmuch as until actual infection has occurred tuberculosis can have no existence in the individual, but the slight manifestations which have been grouped under this head really comprise the earliest symptoms of the disease itself.

This is the golden opportunity, the time of all times to make the diagnosis, because now the patient's prospects for recovery are most excellent; indeed, they are far better than they can ever be at any subsequent period.

But why is this opportunity not more frequently recognised and taken advantage of by the physician? In the first place, because of the apparent insignificance of the symptoms which attend the onset of the truly early stage, and which the patient is prone to neglect as of not sufficient importance to warrant professional consultation. The malaise, loss of appetite and slight decline in weight which he may experience, he readily attributes to all sorts of supposed minor aliments, for he does not know that these symptoms may mean the advent of so serious a disease as tuberculosis. For the patient's neglect of himself the profession is not responsible, but we may perhaps do something toward educating the public to the realisation of the fact that such manifestations are often worthy of a more earnest consideration and that an investigation as to their cause is always justifiable. While it is, of course, true that the cause may, in numerous instances, prove an innocent one, a careful investigation can, on the one hand, do no harm, whereas on the other, neglect may lead to fatal consequences in the end. Again, it is the general practitioner, the family doctor only, and not the specialist, to whom opportunity is afforded at all frequently to recognise these suggestive symptoms.

The knowledge that tuberculosis has existed in a family, the casual observation that certain members of a family appear less robust than others, that one child is retarded in its physical growth and development as compared with its brothers and sisters, and the like, should all serve as warnings to render him

1 H. P. Loomis, Transactions Am. Clim. Association, Vol. XIV., 1898, p. 49.

alert and watchful to detect the very earliest indications of a tuberculous affection. In this connection it seems almost superfluous to speak of the advisability of subjecting to repeated examinations, from time to time, the relatives of phthisical subjects, whose close relations in nursing and caring for the invalid and in whom mere domiciliary contact may render infection liable. As proof of the practical value of adopting such a method of procedure, I may add that it has been by no means an infrequent experience in the Winyah Sanitarium to find on examination of relatives, accompanying phthisical patients who have entered the institution for treatment, evidences of more incipient tuberculous processes, the existence of which had never been suspected.

It is not in every case, however, that the failure to obtain an early diagnosis is due to the patient's neglect to seek medical advice. Too often the physician also is found wanting. He may allow himself to be influenced by the patient's own statements and agree with the latter that the slight symptoms of which he complains depend upon overwork or some unimportant ailment; again, in the hurry of a general practice the physician frequently satisfies himself with the advice to take a vacation or with the prescription of a simple tonic and neglects to institute a careful and thorough physical examination. Such action was perhaps excusable in past times when the opinion was uniformly held that tuberculosis was an incurable disease, and when the diagnostic means at hand were not so thoroughly exploited as they are in these days of modern scientific progress. But times have changed and with a more exact knowledge of the special pathology of tuberculous affections, and with the rapidly growing interest in such conditions, particularly during the last decade, we have arrived at a better understanding of all that pertains to tuberculosis. We have learned that this disease is curable if taken in time and consequently the paramount importance of its early recognition has come to appeal more and more strongly to us as a profession.

Now, what are the physical signs and subjective symptoms which we must seek to recognise if we will make an early diagnosis in the true sense of the word; that is, at that period in which the first invasion of tubercles occurs in the lungs and before destructive changes are already conditioned or impending?

Among the symptoms which the patient himself may experience may be enumerated slight elevation of temperature, loss of appetite, decline in weight, malaise, and occasionally digestive disturbances. The rise of temperature during the stage of tubercle formation rarely exceeds 99° to 100° F., and of this the patient is usually quite unconscious, for he soon becomes accustomed to it. For this reason it is essential to depend entirely upon the thermometer and to make a careful temperature record at two hourly intervals. A temperature disturbance, such as has been indicated, particularly if it is accentuated by physical exercise or occurs after meals or, again, in women if it becomes more marked at the menstrual period, together with part or all of the subjective symptoms which have been mentioned, is highly suggestive, and should cause the physician to institute a thorough and painstaking physical examination without delay.

It may possibly be noted that cough has not been mentioned as one of the early symptoms. I have purposely omitted it thus far because I desire to direct attention to the fact that it is by no means a constant phenomenon in the incipient stage of pulmonary tuberculosis. When cough is present it is due to catarrh of the alveoli or bronchioles or of both, but in cases in which the catarrhal changes are slight and little or no secretion enters the bronchioles, there may not be sufficient stimulus to induce it; or, again, it may be produced only by deep inspirations during physical exertion, laughing, talking, and the like. Consequently we must not be led by the absence of cough to indulge in a false sense of security.

Pleuritic pains and soreness in the chest, while only occasionally early symptoms, should not be overlooked. It is true that quite often inflammatory conditions of the pleura antedate demonstrable changes in the lungs by months and years. On the other hand, many persons who have suffered from pleurisy never show evidence of pulmonary tuberculosis at subsequent periods. At the same time, however, it appears to be well-established that the so-called idiopathic pleurisies which are attributed to exposure to cold, and the like, have no existence in fact, no more than do inflammations of other serous membranes, as those of joints and the peritoneum. Such inflammations, there is ample reason to believe, are due to microbic infection, and while a large variety of pathogenic microörganisms may give rise to these conditions it has been proven that very frequently their nature is tuberculous. The investigations of Schlenker showed that 61 out of 85 cases of pleurisy which he examined were tuberculous, and various other authorities have made similar reports. With such evidence of the frequency of tuberculous involvement of the pleura, all cases of the so-called idiopathic variety, as well as all others in which other etiological factors can not be demonstrated, should be looked upon as in all probability tuberculous until the contrary is proven, while pain in the pleura should always lead the physician to make a careful physical examination. Thus we may discover an otherwise unsuspected tuberculous process of the lung which, but for the warning afforded by the pain, might have progressed for some time without becoming recognised.

Now as to the physical signs of the invasion of tubercles in the lung; in other words, of the incipient stage of pulmonary tuberculosis. At this time there is no percussion dulness, for the eruption of tubercles is not so dense, nor are there present sufficient connective tissue changes, neither is there as yet such obstruction in the alveoli and bronchioles as to produce it. As a matter of fact during this period there is so little evidence to be obtained by percussion that it may practically be ignored as an aid in making the diagnosis.

On auscultation, nevertheless, certain delicate signs are to be detected by the trained ear of the diagnostician. There is to be heard a slightly rough or rough vesicular inspiration, which may be of the interrupted or so-called "cog-wheel" type, independently of transmitted systolic cardiac impulses. At times an extremely delicate apical catarrh may be detected which makes itself appreciable to the auscultator by a slight sense of moisture or "stickiness" and which may attain to the degree of fine crepitation. This latter sign may often be rendered distinct by causing the patient to cough, after which it is to be heard at the end of the next deep inspiration.

The delicate auscultatory phenomena which have been recounted are those of local congestion of the alveoli and in themselves do not give the cue to what etiological factors are responsible. If the signs observed are due to transient conditions, such as colds, capillary bronchitis, and the like, they will gradually diminish and disappear. But, if after repeated examinations at intervals, they are found to *persist over a circumscribed area* of the lung, especially an apex in an adult, there need be no hesitancy in interpreting them as corroborative evidence of the eruption of tubercles.

Furthermore, when the subjective symptoms and perhaps the personal or family history, particularly, as to possible exposure to infection, stand in accord with persistence of the early physical signs mentioned, we should make a positive diagnosis of pulmonary tuberculosis. Such evidence is conclusive and any additional diagnostic means, as for example the tuberculin test, are entirely superfluous. Cases will, of course, present themselves in which there may still exist in the mind of even the most expert diagnostician, an element of doubt as to the true state of affairs, and in which the suspicion of tuberculosis still appears strongly justified. In such instances and also if the physician is not quite positive of his ability to detect the delicate auscultatory phenomena of the formation of new tubercles in the lung, recourse may always be had to tuberculin diagnosis. Herein we have a method which, when properly applied, is not only perfectly safe and free from undesirable effects, but is at the same time eminently reliable as a means of determining beyond the shadow of a doubt whether or not a tuberculous process exists in the suspected individual.

The Röntgen ray has numerous advocates as a diagnostic aid, but its natural limitations must exclude it as affording no information in the period of the first eruption of tubercles. Not until the pathologic changes have advanced to such a degree that areas of considerable consolidation are present can we expect to obtain shadows which can in any way be characteristic. Again, as regards anomaly in the comparative range of movement of the two sides of the diaphragm, which has often been observed in phthisis, since this is due to structural alterations in the affected lung, we certainly cannot afford to wait, if we hope to make an early diagnosis, until such changes have taken place. We might with just as much reason delay in our diagnosis until the advent of the tubercle bacillus in the sputum, and we know that until destructive changes are occurring with softening, liquefaction and evacuation of caseous foci, the appearance of the germ in the expectoration is a manifest impossibility. Besides, an *x*-ray outfit is an expensive apparatus which but a comparatively few practitioners are able or willing to afford, and whose practical employment entails considerable experience to obtain anything like reliable results as a means of diagnosticating even early phthisis.

The method of early diagnosis by sero-agglutination, as practised by Arloing and Courmont, while undoubtedly of value in the hands of experts, implies the possession of facilities as well as a minuteness of technical detail which render it unacceptable to the general practitioner.

A great many other signs and symptoms have been brought forward from time to time as aids in diagnosis. In passing may be mentioned the so-called "pretuberculous albuminuria" of Tessier,[1] of which there is yet not sufficient evidence that it is anything more than an ordinary transient albuminuria, a widely dilated state of the pupils[2], a peculiar facial expression which has been considered as characteristic, abnormal conduction of heart sounds which is, as a rule, indicative of consolidation, a sense of vibration in the chest on speaking or vocal fremitis, of which the patient is himself conscious (which if present is again an evidence of consolidation), lack of lustre of the hair, the well-known clubbing of the nails, and even the odor of the breath, as well as others too numerous to enter into consideration in a paper of limited extent. While such phenomena are of clinical interest and of more or less confirmative diagnostic value, many of them are concomitants of more advanced stages of the disease and none of them can have more than an incidental bearing upon the question of diagnosis, particularly of early diagnosis.

For really practical purposes we must continue to depend for the early recognition of pulmonary tuberculosis, upon the symptoms and usual methods of physical examination in general, and the tuberculin test when in doubt—means which are surely quite sufficient when painstakingly and intelligently exploited.

1. Tessier. Cited in American Year Book of Medicine and Surgery, 1897, p. 35.

2. Thos. F. Harrington, Journal of Tuberculosis, Vol. XI., p. 5.

